# Updates from the TP53 universe

**DOI:** 10.1038/cdd.2017.190

**Published:** 2017-11-10

**Authors:** Francesca Pentimalli

**Affiliations:** 1 Oncology Research Center of Mercogliano (CROM), Istituto Nazionale Tumori – IRCCS -Fondazione G, Pascale, Naples, Italy

TP53 is the most frequently mutated gene in cancer and its study helped to define fundamental principles underlying tumorigenesis. However, despite over three decades of accumulating knowledge (Figure 1), we still have many open questions concerning most facets of p53 functions that hamper our ability to translate p53 knowhow into therapeutic strategies. In this issue of *Cell Death & Differentiation* leading scientists in the p53 field elucidate some of these questions taking us a step ahead towards our understanding of this versatile protein.^1^

P53 controls the expression of wide gene sets owing to its transcriptional activity leading to both mRNA up- and downregulation. However, its ability to act as a direct repressor of transcription has been long disputed. Here Espinosa and colleagues provide evidence that such repressive effects are mainly attributable to downstream effectors such as microRNAs or p21 (encoded by *CDKN1A*) or association with MDM2 – the main p53 regulator – which acts as a repressor by its own.^2^ In particular, as shown by Engeland in this Issue, p21 connects the p53 pathway with the DREAM transcriptional repressor complex which includes members of the retinoblastoma family of tumor suppressors – whose inclusion within the DREAM is regulated by the cyclin-dependent kinases that are inhibited by p21. The DREAM controls hundreds of cell cycle-associated targets, further expanding the number of genes affected by p53 modulation, and connecting p53 to the regulation of all cell cycle checkpoints.^3^ Espinosa also discusses findings further detailing the functions of p53 trans-activation and carboxy terminal domains required for its transcriptional activator role.^2^ Interestingly, p53 emerged as a pioneer transcription factor able to bind a core set of enhancers even when the chromatin is closed, overriding epigenetic regulatory landscapes. Not all of the p53 binding, however, seems productive: rather, it becomes functional upon changes of the chromatin environment, which are time and context dependent. This explains why, although p53 binding to chromatin is largely invariable, the p53-regulated programme is extremely variegated.^2^

Differently from other tumor suppressors that are mostly inactivated by deletions or truncating mutations, *TP53* mainly presents missense mutations, which occur throughout the coding sequence but are predominant in the region encoding the DNA binding domain. The predominance of mutations, rather than wt *TP53* loss, infers a potential selective advantage of such mutants in promoting cancer. But what leads to *TP53* hot-spot mutations and what factors contribute to selection of such specific mutants in cancer? Levine and colleagues here address this question and put forward different hypotheses. They first consider the possibility that the most frequent missense mutations reportedly affecting the p53 DNA binding domain might induce a more denatured protein structure thereby impairing p53 ability to bind DNA. Through a recently developed computational tool, aimed at predicting the outcome of deleterious protein variants by sequence analysis and structural modeling, they show however that most missense mutations give rise to proteins that are structurally similar to wt p53. Conversely, mutations in the 10 top frequently mutated sites fall into two groups: one of proteins structurally unfolded and one of folded but unable-to-bind-DNA proteins (DNA contact mutants). So, they suggest that these two types of mechanisms are the main contributors to the loss of function phenotype characterized by impaired DNA binding and transcriptional ability, which blunt p53 tumor suppressor activities.^4^ Some of the most common missense mutations are likely due to the effect of environmental mutagens, such as UV rays or cigarette smoke, which act in a tissue-specific manner, whereas methylation of CpG residues within the *TP53* sequence seems a more general mechanisms increasing p53 mutational rate in most tissues, which also explains while arginine residues (encoded by four CG-containing codons) are among the top most frequently mutated. The reason why only specific mutations, over all the methylated CpG residues within the *TP53* sequence, are selected could depend on the structural and biological relevance of the affected residues and their contribution in fostering tumorigenesis.^4^

Beyond loss of function, p53 mutants can drive tumorigenesis through other mechanisms: in heterozygosity, when both the wt and mutant alleles coexist, the mutant proteins can act as dominant negative (DN) over the wt, forming heterotetramers with impaired transcriptional ability.^5^ Also, p53 mutants that acquire oncogenic activity through a gain-of-function (GOF) mechanism have an increasingly recognized role. Kim and Lozano in this issue explore a wide set of p53 mutants and the mechanisms whereby they gain new oncogenic functions. Such mutants mostly act through protein-protein interactions with other transcription or chromatin remodelling factors affecting vast downstream gene networks. These interactions, which so far have been mostly described in cell lines, are highly context dependent determining various degrees of GOF activity in different tumor types with mutated p53. Interestingly, it seems that some p53 mutants acquire similar GOF activities binding common transcription factors and transactivating the same gene targets. This could have translational relevance because it would ideally limit the set of targets which should be tackled to suppress the new oncogenic GOFs.^5^ Overall, determining the mechanisms whereby p53 loss of function, DN and GOF activity drive tumor development and progression is crucial to definitively establish the tumor suppressor function of p53.

Induction of apoptosis, cell cycle arrest and senescence are the ‘classical’ outputs of p53 activation that seem to guarantee tumor suppression.^6^ In particular, as discussed in this Issue by Strasser and colleagues, the ability of p53 to induce apoptosis in transforming cells through the direct activation of pro-apoptotic proteins (such as PUMA and NOXA) was initially regarded as the main cancer preventing function of p53. However, in contrast with *Trp53* null mice that develop tumors with complete penetrance within the first year of life, mice depleted of all the (known) main downstream effectors of p53-mediated apoptosis, do not develop spontaneous tumors, even when coupled to the loss of p53 G1/S cell cycle arrest and senescence effectors, suggesting that full p53 antitumoral potential relies on further capabilities.^6, 7^

So, how does p53 really protect from cancer development? Kaiser and Attardi, provide some clues derived from the analysis of genetically engineered mice modelling p53 alterations, which help to reconcile older and new studies. The type of stress that challenges p53 action seems to be the key: whereas the above-mentioned classical outputs of p53 activation are needed to respond to acute DNA damage cues, incipient cancer cells are more likely exposed to other stresses such as nutrient and oxygen deprivation, oncogenic signalling, replication or oxidative stress, and also low levels of genotoxic, chronic DNA damage. To face these, p53 might induce common outcomes (cell death, senescence, differentiation) through alternative routes – triggering metabolic changes, autophagy, promoting DNA repair – in a context-dependent manner. Here the authors analyze both cell autonomous and non-autonomous emerging functions of p53 and its downstream targets, which also play a part in the p53 antitumoral response.^6^

But p53 does not act alone as commander in chief when it comes to cell fate decisions. The fine tuning of the cellular responses to a wide range of stimuli is achieved through the cross-talk between complex gene networks and signalling pathways. As an example, Oren and colleagues here discuss the cross talk between the p53 and the Hippo tumor suppressor pathway, which – similarly to p53 – regulates a variety of cellular processes. The authors highlight how the direct interaction between core components of both p53 family and Hippo pathways, and their regulation of common targets, is coordinated to modulate the response to different stresses and maintain homeostasis between stemness and differentiation, cell death and proliferation, anabolism and catabolism. Alterations of either pathway, as occurring in cancer cells, disrupt this balance with profound effects on cell fate.^8^

Regardless of the mechanism, p53 tumor suppressor functions protect organisms against cancer development by ensuring genome stability through DNA repair or eliminating damaged cells that might undergo neoplastic transformation. However, factors that limit indefinite proliferation protect from cancer but foster aging, the gradual process of cell and tissue deterioration. Depletion of functional cells, stem cell exhaustion and the induction of a chronic inflammatory microenvironment by senescent cells, all feature aging. Although intimately related, the link between p53 and aging is still unclear with p53 activity being found associated to both premature aging and increased longevity. To clarify this conundrum Wu and Prives analyzed the mechanisms whereby p53, and its main regulator MDM2, could affect aging by regulation of genomic stability, senescence, cross talk with metabolic pathways (such as the insulin/IGF1 and mTOR pathways), mitochondrial dysfunction and regulation of reactive oxygen species, at the cellular level. Also, based on the analysis of mouse models with modulated p53-MDM2 axis, they propose a model to infer the relation between lifespan and cancer resistance as a function of p53 stability achieved through the loss of MDM2 regulation or chronic stress. Hyperactivation of p53 could provide greater cancer resistance but at the cost of accelerated ageing. Therefore, the underlying mechanisms of this delicate balance will have to be carefully determined when considering anti-aging approaches as well p53-reactivating strategies against cancer.^9^

Another layer of complexity, hampering our understanding of p53 physiological and pathological functions is added by the roles, overlapping or disjointed, of the other family members p63 and p73 and their respective isoforms. The presence of an highly conserved DNA binding domain shared by the three members of the p53 family explains some functional redundancy in their regulation of gene expression. However, p63 and p73 can also forms heterotetramers that are more stable than individual tetramers. Indeed both proteins are found to co-occupy DNA target sites throughout the genome, as discussed here by Melino and colleagues, who argue that studying these genes singularly could provide only a restricted view of their global functions. Melino and colleagues also report on latest findings implicating p63 and p73 in the regulation of cell metabolism which also contributes to the p53-family-mediated flexible response to stress. Besides tumor-related functions the authors present recent evidence establishing p73 as the master regulator of ciliogenesis whose defects underlie various human diseases. This overlooked function finally explains disparate multi-organ defects previously observed in p73 knock out models providing a unifying view that will help to better characterize other non-oncogenic functions of p73 such as infertility neurodevelopment and inflammation.

## Figures and Tables

**Figure 1:**
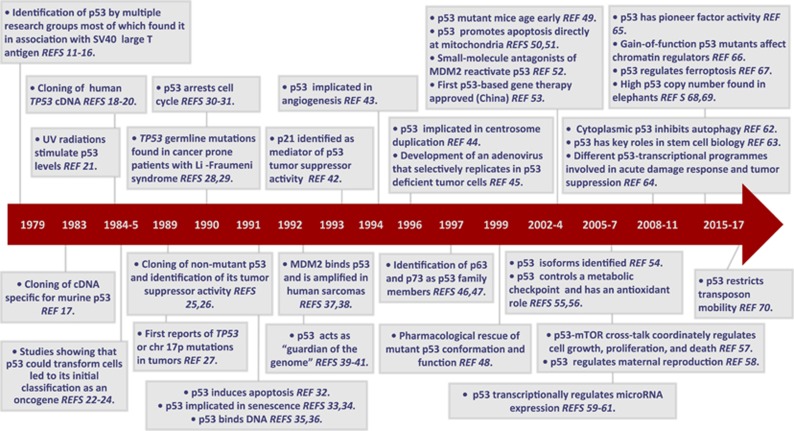
Timeline of breakthrough discoveries achieved since p53 identification. Key milestones are indicated amongst all the studies that contributed to define p53 functions. Many other landmark studies, such as those reporting the roles of the other family members, studies in other species and particularly those in mouse models, as well as many others, are not reported owing to space constraints
